# How best to design a clinical trial that is relevant to practice? Applying PRECIS-2, a trial design tool, to primary care trials

**DOI:** 10.1186/1745-6215-16-S2-P214

**Published:** 2015-11-16

**Authors:** Gordon Forbes, Kirsty Loudon, Shaun Treweek, Stephanie Taylor, Sandra Eldridge

**Affiliations:** 1 Queen Mary University London, London, UK; 2 University of Aberdeen, Aberdeen, UK; 3 University of Dundee, Dundee, UK

## 

For results from a randomised trial to be relevant to practice it is important that the needs of the end users of the trial, health care professionals, policymakers and patients are considered. Key aspects of a trial need to reflect how the intervention would be implemented. PRECIS-2 is an innovative way to display information about a trial's design, using a wheel with 9 spokes or domains, graphically illustrating which areas of the trial are more pragmatic, that is designed to test whether the intervention will work in usual care and which are more explanatory, designed to test the intervention under ideal conditions.

This talk will describe the results of a two-part investigation applying PRECIS-2 to primary care trials. In the first part we worked with primary care trial teams to apply the tool to their trials. In the second part we interviewed people involved in trying to change practice based on research evidence to find out their views on PRECIS-2 and what is important to ensure the results of trials are relevant to practice. The interviewees included research funders, guideline developers and practicing GPs.

We found that primary care trials vary considerably in the extent to which they can be viewed as pragmatic, that PRECIS-2 addresses aspects of trial design which policy makers, practitioners and guideline developers believe are important in assessing relevance to practice and that for some areas of a trial's design flexibility should not always be encouraged. Our interviewees also suggested some novel uses for PRECIS-2.

